# Population-based incidence rate of in-hospital delirium in older adults in Norway—a HUNT study

**DOI:** 10.1093/ageing/afag297

**Published:** 2026-10-11

**Authors:** Wilhelm Frich, Kristine Ringnes Verlo, Miguel Germán Borda, Thomas Andrew Jackson, Gill Livingston, Christian Myrstad, Kenneth Rockwood, Geir Selbæk, Håvard Kjesbu Skjellegrind, Bjørn Heine Strand, Gro Gujord Tangen, Leiv Otto Watne, Yehani Wedatilake, Dag Årsland, Bjørn Erik Neerland

**Affiliations:** Oslo Delirium Research Group, Institute of Clinical Medicine, University of Oslo, Oslo, Norway; Department of Geriatric Medicine, Oslo University Hospital, Oslo, Norway; Oslo Delirium Research Group, Institute of Clinical Medicine, University of Oslo, Oslo, Norway; Department of Geriatric Medicine, Oslo University Hospital, Oslo, Norway; Centre for Age-Related Medicine (SESAM), Stavanger University Hospital, Stavanger, Norway; Department of Neurology, Clínica Universidad de Navarra, Pamplona, Spain; Department of Inflammation and Ageing, Centre for Translational Inflammation Research, Queen Elizabeth Hospital, Mindelsohn Way, University of Birmingham, Birmingham B15 2WD, UK; Division of Psychiatry, University College London, London, England, UK; St. Pancras Hospital, North London NHS Foundation Trust, London, UK; Department of Medicine, Levanger Hospital, Nord-Trøndelag Hospital Trust, Levanger, Nord-Trøndelag, Norway; Department of Medicine, Dalhousie University, Halifax, Nova Scotia, Canada; Nova Scotia Health Frailty and Elderly Care Network, Halifax, Nova Scotia, Canada; Oslo Delirium Research Group, Institute of Clinical Medicine, University of Oslo, Oslo, Norway; Department of Geriatric Medicine, Oslo University Hospital, Oslo, Norway; Norwegian National Centre for Ageing and Health, Vestfold Hospital Trust, Tønsberg, Norway; HUNT Research Centre, Department of Public Health and Nursing, Norwegian University of Science and Technology, Faculty of Medicine and Health Sciences, Levanger, Norway; Levanger Hospital, Levanger, Nord-Trøndelag Hospital Trust, Nord-Trøndelag, Norway; Department of Geriatric Medicine, Oslo University Hospital, Oslo, Norway; Norwegian National Centre for Ageing and Health, Vestfold Hospital Trust, Tønsberg, Norway; Department of Physical Health and Ageing, Norwegian Institute of Public Health (NIPH), Oslo, Norway; Department of Geriatric Medicine, Oslo University Hospital, Oslo, Norway; Norwegian National Centre for Ageing and Health, Vestfold Hospital Trust, Tønsberg, Norway; Oslo Delirium Research Group, Institute of Clinical Medicine, University of Oslo, Oslo, Norway; Department of Geriatric Medicine, Akershus University Hospital, Lørenskog, Akershus, Norway; Department of Geriatric Medicine, Oslo University Hospital, Oslo, Norway; Norwegian National Centre for Ageing and Health, Vestfold Hospital Trust, Tønsberg, Norway; Centre for Age-Related Medicine (SESAM), Stavanger University Hospital, Stavanger, Norway; Centre for Healthy Brain Ageing, Department of Psychological Medicine, Institute of Psychiatry, Psychology, and Neuroscience, King's College London, London, UK; Oslo Delirium Research Group, Institute of Clinical Medicine, University of Oslo, Oslo, Norway; Department of Geriatric Medicine, Oslo University Hospital, Oslo, Norway; Norwegian National Centre for Ageing and Health, Vestfold Hospital Trust, Tønsberg, Norway

**Keywords:** delirium, incidence, epidemiology, population-based study, chart review, older people

## Abstract

**Background:**

Most studies of delirium occurrence are based on selected hospitalised populations or administrative data. We aimed to estimate the incidence rate of in-hospital delirium in older adults and to assess use of the International Classification of Diseases, 10th Revision (ICD-10) delirium code (F05) in electronic health records.

**Methods:**

This population-based cohort study included 9956 participants aged ≥70 years from HUNT4 70+. Participants were followed from the date of study participation (2017–19) until 31 December 2023. In-hospital delirium was identified by retrospective chart review of all admissions during follow-up, using Diagnostic and Statistical Manual of Mental Disorders, fifth edition criteria. Incidence rates were estimated using the first delirium episode per participant, weighted for non-participation.

**Results:**

During follow-up, 1507 participants (15.1%) experienced ≥1 in-hospital delirium episode. The weighted incidence rate was 33.1 (95% CI 31.5–34.9) per 1000 person-years and increased with age from 11.4 (age 70–74) to 109.0 (age ≥90) per 1000 person-years. Men had higher incidence rates than women across age groups. Among admissions with delirium, 11.9% had an ICD-10 discharge delirium diagnosis code (F05).

**Conclusion:**

In-hospital delirium was common, increased markedly with age and was rarely captured by diagnostic coding. Low coding rates suggest that routine health-care data substantially underestimate delirium burden. Under-recognition of delirium may limit opportunities for follow-up in a group at high risk of adverse outcomes. With an ageing population, the burden of delirium is likely to increase for individuals and health-care systems. Our findings support systematic approaches to improve identification and management.

## Key points

- In-hospital delirium was common among older adults in this population-based cohort, but rarely captured by diagnostic coding.
- Incidence rates increased approximately tenfold with age and were higher in men than in women.
- Better recognition of delirium may support clinical follow-up in a group at high risk of adverse outcomes.
- Reliable incidence estimates can facilitate planning future health service needs and inform strategies for delirium prevention.

## Introduction

Delirium is an acute neuropsychiatric syndrome characterised by altered attention, awareness and cognition [1]. It can be triggered by acute illness, surgery, medication effects or other physiological disturbances, particularly in individuals with predisposing factors such as older age, dementia and frailty [2]. Delirium can be highly distressing for patients and relatives and is associated with adverse outcomes, including increased mortality, care home admission and higher economic costs per hospital admission [3, 4]. It has also been associated with an increased risk of future dementia and worsening of pre-existing dementia [5, 6]. Multicomponent interventions have been shown to reduce the risk of delirium [7].

Most evidence on delirium occurrence comes from hospitalised patients. In medical inpatients, prevalence has been estimated at 23%, with higher prevalence in high-risk settings such as geriatric wards and intensive care units [1, 8]. Population-based incidence estimates of in-hospital delirium are scarce. Studies relying on hospital discharge diagnoses or administrative coding have reported incidence rates of 1–5 per 1000 person-years [9, 10], but diagnostic coding captures only a minority of delirium episodes [11].

In a Norwegian community-based cohort of older adults (*n* = 2115), Titlestad *et al.* reported an incidence rate of 29.8 per 1000 person-years based on chart review, compared with 3.4 per 1000 person-years from discharge diagnoses [9]. This highlighted the limitations of discharge diagnoses for estimating delirium occurrence. Population-based incidence estimates from larger, well-characterised cohorts remain limited. The Delirium and Population Health Informatics Cohort (DELPHIC) study in the United Kingdom (UK) provided prospective data on in-hospital delirium in 1510 community-dwelling older adults, but did not report incidence rates per person-year [12].

In contrast to cumulative incidence, incidence rates per person-year account for events and observation time and can therefore provide a more informative description of delirium occurrence in studies with loss to follow-up or mortality. With an ageing population and increasing dementia prevalence, reliable estimates of delirium incidence in the general older population are needed. Such estimates can facilitate planning for future health service needs and inform strategies for delirium prevention.

The aim of this study was to estimate the incidence rate of in-hospital delirium in a large population-based cohort of older adults, overall and by age group and sex. In addition, we aimed to assess the use of the International Classification of Diseases, 10th Revision (ICD-10) code for delirium (F05) among identified episodes.

## Methods

### Study design and participants

This population-based cohort study included participants from HUNT4 70+, a sub-study of the Trøndelag Health Study (HUNT) in Norway [13]. All inhabitants aged ≥70 years in the Nord-Trøndelag region were invited, with no additional inclusion or exclusion criteria. Nord-Trøndelag is broadly representative of Norway in terms of cause-specific mortality and overall health, but has fewer immigrants and individuals with higher education [14].

Baseline was defined as the date of HUNT4 70+ participation between September 2017 and March 2019. Baseline data were collected at field stations, in participants’ homes, or in nursing homes. Of 19 403 invited individuals, 9956 (51.3%) participated (Figure 1).

**Figure 1:**
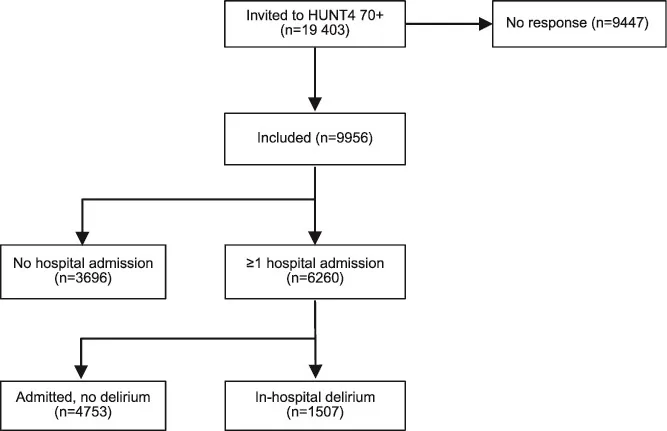
Flow chart. Participants were followed from HUNT4 70+ participation (2017–19) to 31 December 2023.

HUNT4 70+ included questionnaires, clinical examinations and cognitive and physical testing [13]. Cognitive status (no cognitive impairment, mild cognitive impairment or dementia) was determined by an expert panel based on test results and interviews with participants and informants [15]. Frailty was assessed using the HUNT4 frailty index (HUNT4-FI) [16]. Sex was obtained from national registers. Education was self-reported and categorised as primary (<10 years), secondary (10–12 years), or tertiary (>12 years), supplemented with HUNT2 or HUNT1 data if missing.

This study was approved by the Regional Committee for Medical and Health Research Ethics in Norway (REK 592609/715718).

### Delirium assessments

In-hospital delirium was defined as delirium present at any time during hospital admission, regardless of onset. All overnight somatic hospital admissions between 1 January 2006 and 31 December 2023 were reviewed as part of a larger HUNT delirium project. For the present incidence analyses, follow-up started at HUNT4 70+ participation (2017–19), and only admissions occurring after baseline were included.

Delirium was assessed through systematic retrospective chart review using the Diagnostic and Statistical Manual of Mental Disorders, fifth edition (DSM-5) criteria (Appendix S10) [17]. Electronic health records (EHRs) were available from both local hospitals in Nord-Trøndelag and the regional university hospital, capturing virtually all admissions in the region.

Chart reviews were conducted by eleven raters, including physicians with experience in geriatrics, a nurse and medical students. Raters were trained in delirium diagnosis, DSM-5 criteria, and chart review methodology, and were blinded to all HUNT data. Each admission was reviewed by one rater. Uncertain cases were discussed with one of three physicians experienced in geriatrics and delirium, and regular meetings were held to resolve difficult cases.

For each admission, all available EHR documentation was manually reviewed, and symptoms corresponding to DSM-5 criteria were recorded in an electronic case report form (eCRF). When explicit documentation of impaired attention was lacking, altered awareness was accepted as the primary indicator of DSM-5 criterion A (Appendix S9).

Because delirium was assessed retrospectively and diagnostic uncertainty was expected, each admission was classified into three categories: probable, possible and no delirium. *Probable delirium* required fulfilment of all five DSM-5 criteria based on chart review, with criterion E assumed to be fulfilled for all included somatic hospital admissions (Appendix S9). *Possible delirium* required fulfilment of three or four criteria, with overall documentation supporting delirium. All remaining admissions were classified as *no delirium*.

In the following, ‘delirium’ refers to admissions classified as probable delirium, unless otherwise specified.

Additional information recorded included admission type, hospital department, explicit mention of ‘delirium’ in the EHRs, and coding of delirium (F05) in the discharge summary. Inter-rater reliability for the three-category delirium classification was assessed in two rounds and showed substantial to almost perfect agreement (weighted Fleiss’ kappa 0.65 and 0.80).

Additional details on DSM-5 operationalisation, motor subtype classification, the eCRF and inter-rater reliability are provided in Appendices S9 and S11.

### Statistical analysis

Descriptive statistics are presented overall and by in-hospital delirium status (≥1 episode).

Participants were followed from HUNT4 70+ participation until first delirium episode, death, or 31 December 2023, whichever occurred first. Only the first delirium episode per participant was included in the incidence analyses, as the primary aim was to estimate incidence rates of first in-hospital delirium rather than recurrent episodes.

Because only the month of death was recorded, the date of death was set to the last day of the recorded month to avoid censoring delirium events later that month. In a sensitivity analysis, participants who died without prior delirium were instead censored on the 15th of that month.

Person-time included all follow-up time, regardless of hospitalisation. Incidence rates per 1000 person-years were calculated using the first delirium episode per participant and total person-time at risk. For inverse probability weighting (IPW)-weighted rates, 95% confidence intervals (CIs) were estimated using jackknife methods.

To allow participants to contribute person-time to different age groups, follow-up was split into 5-year age bands using a Lexis approach.

To account for non-response and healthy volunteer bias, we applied IPW using previously published weights based on age, sex and education [18]. Weights were also calibrated for nursing home residence because nursing home residents were slightly overrepresented compared with the Nord-Trøndelag population in 2019 (6.3% vs 5.1%). Participants with missing education data (*n* = 85, 0.9%) were excluded from the primary weighted analyses.

Absolute differences in incidence rates between men and women were estimated using weighted Poisson regression models with person-time as the exposure. Marginal predictions provided rate differences with 95% CIs, and robust standard errors accounted for multiple time intervals per individual.

Among delirium episodes, we calculated the proportion with an ICD-10 delirium code (F05) and with explicit documentation of ‘delirium’ in the EHR. Sensitivity analyses included: (i) inclusion of both possible and probable delirium episodes in the primary outcome; (ii) exclusion of participants living in nursing homes at baseline; and (iii) exclusion of participants with dementia at baseline. To provide context for the incidence estimates, we described crude hospital admission rates by age group and sex as a supplementary analysis.

There was no formal pre-specified analysis plan, but the statistical methods for the incidence analyses were agreed before the final dataset was available. This study was reported in accordance with the STROBE Statement for cohort studies (Appendix S12). Analyses were performed in Stata 19, except for inter-rater reliability analyses, which were performed in Stata 18.

## Results

The 9956 participants contributed 47 993 person-years. Median follow-up was 5.5 years (IQR 4.7–6.0). Mean age at baseline was 78.1 years (SD 6.5) and 54.4% were women. Participant characteristics are shown in Table 1.

**Table 1:** Baseline characteristics at HUNT4 70+ participation overall and by incident delirium status (≥1 episode between HUNT4 70+ and 31 December 2023).

	All	Delirium (≥1 episode)	No delirium
Number of participants	9956	1507 (15.1)	8449 (84.9)
Age, years—mean (SD)	78.1 (6.5)	81.8 (6.8)	77.4 (6.2)
BMI, kg/m^2^—mean (SD)	27.2 (4.3)	26.9 (4.6)	27.2 (4.4)
**Sex**			
Male	4536 (45.6)	744 (49.4)	3792 (44.9)
Female	5420 (54.4)	763 (50.6)	4657 (55.1)
**Test location**			
Field station	8522 (85.6)	1050 (69.7)	7472 (88.4)
Home visit	803 (8.1)	308 (20.4)	495 (5.9)
Nursing home	631 (6.3)	149 (9.9)	482 (5.7)
**Education**			
Primary	3199 (32.1)	606 (40.2)	2593 (30.7)
Secondary	4293 (43.1)	620 (41.1)	3673 (43.5)
Tertiary	2379 (23.9)	251 (16.7)	2128 (25.2)
Missing	85 (0.9)	30 (2.0)	55 (0.7)
**Frailty (HUNT4-FI) category**			
Robust (<0.15)	3099 (31.1)	162 (10.8)	2937 (34.8)
Pre-frail (≥0.15 to <0.25)	3170 (31.8)	369 (24.5)	2801 (33.2)
Frail (≥0.25)	3053 (30.7)	800 (53.1)	2253 (26.7)
Missing	634 (6.4)	176 (11.7)	458 (5.4)
**Cognitive status (HUNT4 70+)**			
No cognitive impairment	4821 (48.4)	403 (26.7)	4418 (52.3)
Mild cognitive impairment	3417 (34.3)	600 (39.8)	2817 (33.3)
Dementia	1531 (15.4)	449 (29.8)	1082 (12.8)
Other or uncertain	187 (1.9)	55 (3.7)	132 (1.6)

Categorical variables are presented as *n* (%), and continuous variables as mean (SD). Percentages are calculated within each column; for the total number of participants, they represent the proportion of the overall cohort. BMI data were available for 9458 participants (95.0%).

During follow-up, 6260 participants (62.9%) had 19 272 hospital admissions, of which 2370 (12.3%) involved delirium. Admission characteristics overall and by delirium status are shown in Appendix S1. Admission rates increased with age and were higher in men than in women (Appendix S2).

Among 2370 delirium admissions, 283 (11.9%) had an ICD-10 delirium discharge diagnosis code, and the term ‘delirium’ was explicitly documented in the EHR in 617 admissions (26.0%). Hypoactive delirium was more common in women than in men and was less often ICD-10 coded than hyperactive delirium (5.8% vs 15.7%; Appendix S4).

Overall, 1507 participants (15.1%) experienced in-hospital delirium during follow-up (Appendix S3). The crude and IPW-weighted incidence rates were 31.4 (95% CI 29.9–33.0) and 33.1 (95% CI 31.5–34.9) per 1000 person-years, respectively. Weighted incidence rates increased markedly with age, from 11.4 (95% CI 9.5–13.8) per 1000 person-years at age 70–74 years to 109.0 (95% CI 97.8–121.6) at age ≥90 years.

Incidence rates were higher in men across age groups, with overall weighted rates of 36.2 (95% CI 33.6–39.0) and 30.6 (95% CI 28.5–32.9) per 1000 person-years, respectively (Table 2 and Figure 2). Absolute rate differences increased with age, although the 95% CI for the difference included zero in the oldest age group (Appendix S5).

**Table 2:** Weighted (IPW) incidence rates of first in-hospital delirium by sex and age group.

Age group	Person-years (events)	Incidence rate	95% CI
**Men**			
70–74	5156 (74)	14.7	11.7–18.7
75–79	8525 (188)	22.2	19.2–25.8
80–84	4780 (186)	39.7	34.3–46.2
85–89	2233 (174)	80.6	69.5–93.9
≥90	1023 (122)	121.8	101.8–146.6
**70+ total**	**21 717 (744)**	**36.2**	**33.6–39.0**
**Women**			
70–74	5445 (45)	8.3	6.2–11.3
75–79	9610 (131)	13.6	11.5–16.3
80–84	5791 (162)	27.7	23.7–32.5
85–89	3361 (210)	61.9	54.1–71.2
≥90	2069 (215)	102.9	90.0–118.1
**70+ total**	**26 276 (763)**	**30.6**	**28.5–32.9**
**Total**			
70–74	10 601 (119)	11.4	9.5–13.8
75–79	18 135 (319)	17.7	15.8–19.8
80–84	10 571 (348)	33.0	29.7–36.8
85–89	5594 (384)	69.2	62.6–76.6
≥90	3093 (337)	109.0	97.8–121.6
**70+ total**	**47 993 (1507)**	**33.1**	**31.5–34.9**

Person-years and events are unweighted (observed). Rates per 1000 person-years. Bold values in the 70+ total rows show totals for person-years and events and the overall incidence rates.

**Figure 2:**
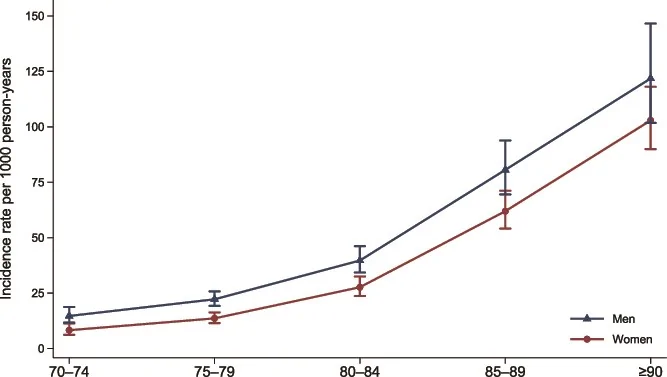
Age-specific incidence rates of in-hospital delirium per 1000 person-years (IPW). Error bars indicate 95% jackknife CIs.

Sensitivity analyses supported the main findings. Including both possible and probable delirium episodes increased the weighted incidence rate to 52.1 (95% CI 50.0–54.3) per 1000 person-years (Appendix S6). Excluding baseline nursing home residents yielded a weighted rate of 31.1 (95% CI 29.5–32.8), while the crude rate among nursing home residents was 117.5 (95% CI 100.0–138.0) per 1000 person-years (Appendix S7). Excluding participants with baseline dementia reduced the weighted rate to 25.8 (95% CI 24.3–27.4) per 1000 person-years (Appendix S8). Censoring participants who died without prior delirium on the 15th of the recorded month produced a virtually unchanged weighted rate of 33.2 (95% CI 31.5–34.9) per 1000 person-years.

## Discussion

In this large population-based study of adults aged 70 years and older, in-hospital delirium was common, affecting 15.1% of participants during a median of 5.5 years of follow-up. Incidence rates increased markedly with age and were higher in men than in women across age groups. Only 11.9% of delirium episodes had an ICD-10 delirium diagnosis code (F05).

The overall incidence rate was broadly consistent with that reported by Titlestad *et al.,* who used retrospective chart review in another Norwegian population-based cohort [9]. We add to this by studying a larger, well-characterised cohort and providing age- and sex-specific incidence rates. In the DELPHIC study, prospective daily assessments during hospital admissions identified in-hospital delirium in 8% of participants over a median follow-up of 2.5 years [12]. Together, these findings indicate that in-hospital delirium is common in older community-based populations, not only in selected hospital cohorts.

The incidence rate increased approximately tenfold from age 70–74 years to age ≥90 years. Several hospital-based studies have reported a pronounced age gradient in delirium occurrence [2, 19]. Higher dementia prevalence likely explains some of the increased vulnerability in older adults. However, in sensitivity analyses excluding participants with dementia, the increase with age remained pronounced, suggesting that factors beyond dementia contribute. Older individuals are also more frequently exposed to acute illness and hospitalisation. However, hospital admission rates increased less markedly with age than did delirium incidence, suggesting that admissions alone may not fully explain the observed age gradient.

Delirium incidence rates were higher in men than in women, with absolute differences increasing with age. Evidence on sex differences in delirium risk is scarce and conflicting, with studies reporting both male and female sex as predisposing factors [20]. The sex-specific rates were stratified by age but not adjusted for other differences between men and women. Higher hospital admission rates in men may partly explain the observed pattern. Differences in dementia prevalence may also be relevant, although higher delirium incidence in men persisted after excluding participants with baseline dementia.

We observed modest differences in the distribution of delirium subtypes: hyperactive delirium was more common in men, whereas hypoactive delirium was more common in women (Appendix S4). Hypoactive delirium may be less likely to be recognised and documented. This may have led to some hypoactive episodes being missed, potentially contributing to the higher observed delirium incidence in men.

In our study, only 11.9% of delirium episodes were captured by ICD-10 coding, consistent with previous evidence of underreporting in administrative data [11]. Coding was substantially less frequent in hypoactive than in hyperactive delirium (5.8% vs 15.7%). Symptoms of hyperactive delirium, such as agitation and restlessness, may be easier to recognise and therefore more likely to be coded. Studies relying on diagnostic coding may therefore overrepresent hyperactive delirium, potentially biasing estimates of both incidence and associated outcomes.

Delirium is increasingly recognised as a sentinel event reflecting underlying vulnerability and increased risk of subsequent adverse outcomes, particularly incident dementia [5, 6, 21]. Under-recognition of delirium may therefore limit opportunities to identify high-risk individuals and initiate appropriate follow-up.

Effective treatment and prevention require identification of patients with delirium and those at risk, as well as accurate and timely information for patients and caregivers. The low levels of explicit documentation and diagnostic coding of delirium in our study support the need for systematic approaches to improve its identification and documentation in clinical practice.

Several guidelines recommend routine delirium screening in hospitalised older adults using brief tools such as the 4 A’s Test (4AT) [22–25]. Penfold *et al.* recently reported recording of 4AT scores on admission for 82.7% of patients ≥65 years at three Scottish hospitals, indicating relatively high adherence to national guidelines [26]. Furthermore, a recent NHS England Pathway recommends delirium screening with 4AT within 30 minutes of arrival in all emergency admissions in patients aged >65 [27]. Economic incentives may also support implementation. In the UK, the introduction of best practice tariffs for hip fracture care, which include preoperative and postoperative delirium assessment, has achieved high adherence [28]. These findings suggest that systematic screening and incentives may improve the identification and documentation of delirium, although continued evaluation of such measures is warranted.

A key strength of the study is the population-based design and large sample size, making it, to our knowledge, the largest population-based study to date with systematic chart-based delirium ascertainment.

Baseline assessments were conducted at field stations, at home or in nursing homes, facilitating inclusion of older and frailer individuals. The overall participation was 51.3%, which is relatively high for this age group, and IPW was used to account for non-response bias and improve representativeness of the Nord-Trøndelag population aged 70 years or older. The HUNT4 70+ cohort is also well characterised, with detailed information on frailty, cognitive status and other health-related factors.

Another strength is the rigorous approach to delirium identification. We manually reviewed all available hospital records and systematically applied DSM-5 criteria. This chart-based approach is more sensitive than reliance on diagnostic coding, as reflected by the low proportion of ICD-10-coded cases in our material. To improve consistency, reviewers held regular meetings to discuss uncertain cases. Inter-rater reliability was assessed and showed high agreement.

A limitation of the study is the retrospective design. Incomplete documentation of signs and symptoms likely resulted in some delirium episodes being missed, leading to underestimation of delirium incidence. The pragmatic use of altered awareness as the primary indicator of DSM-5 criterion A when impaired attention was not explicitly documented may also have introduced diagnostic uncertainty. Distinguishing delirium from conditions with overlapping symptoms, such as dementia or stroke, can also be challenging, particularly when documentation of functional status prior to admission is limited. Prospective bedside assessment using standardised instruments remains the reference standard for delirium diagnosis but is rarely feasible in large population-based cohort studies with long follow-up.

Chart-based methods for delirium identification have acceptable diagnostic accuracy compared with clinical assessments using standardised tools [29, 30]. Some diagnostic uncertainty nevertheless remains. To address this, hospital admissions were classified into three categories (no delirium, possible delirium and probable delirium), reflecting increasing levels of diagnostic certainty. The possible delirium category likely includes a mixture of true delirium episodes with insufficient documentation, conditions with overlapping symptoms and subsyndromal delirium. By restricting the primary outcome to probable delirium, we prioritised specificity and reduced the risk of false-positive classification due to incomplete or ambiguous documentation. The resulting incidence rates are therefore likely to be conservative estimates of the true incidence of in-hospital delirium.

The study did not capture delirium episodes occurring outside hospital. This is particularly relevant for nursing home residents, where acute illness and delirium may be managed without hospital admission. We found an in-hospital delirium incidence rate of 117.5 per 1000 person-years in this group, while a recent review found higher incidence estimates in residential care settings [31]. In our study, the delirium burden of nursing home residents is therefore probably underestimated.

Several individuals experienced recurrent episodes of delirium during follow-up (Appendix S1). The total delirium burden on health-care systems will therefore be higher than our estimated incidence rates. Recurrent delirium episodes are also clinically important, as the risk of subsequent cognitive impairment increases [32].

The study was conducted in one region in a high-income country, and generalisability to other populations may be limited.

In conclusion, in-hospital delirium was common in this population-based cohort of older adults and increased markedly with age, but was rarely captured by diagnostic coding. Under-recognition and under-documentation of delirium may limit appropriate follow-up in a group at high risk of adverse outcomes. With an ageing population, the impact of delirium on patients and health-care systems is likely to increase considerably. Our findings highlight the need for systematic approaches to improve the identification, prevention and management of delirium in clinical practice.

## Supplementary Material

Supplementary_materials_afag297

## Research Data Transparency and Availability

Due to privacy protection for participants, the HUNT Research Centre aims to limit storage of data outside the HUNT databank and cannot deposit data in open repositories. The HUNT databank has precise information on all data exported to different projects and can reproduce these on request. There are no restrictions regarding data export if applications are approved by the HUNT Research Centre. For more information, see: http://www.ntnu.edu/hunt/data
