# Supplementary material for: Population-based incidence rate of in-hospital delirium in older adults in Norway—a HUNT study
Source: Age Ageing. 2026 Oct 11;55(10):afag297. doi: 10.1093/ageing/afag297 (PMC13654089; doi:10.1093/ageing/afag297)
Supplement: Supplementary file 1 [file supplementary_materials_afag297.docx]

**Supplementary data**

**Population-based Incidence Rate of In-Hospital Delirium in Older Adults in Norway – a HUNT study**

**Table of contents**

| **Supplementary item** | **Page** |
| --- | --- |
| **Appendix 1.** Characteristics of all hospital admissions | 2 |
| **Appendix 2.** Hospital admission rates after HUNT4 70+ | 2 |
| **Appendix 3.** First events during follow-up by age group | 3 |
| **Appendix 4.** Distributions of delirium subtypes among probable delirium cases | 3 |
| **Appendix 5**. Age-specific incidence rates of in-hospital delirium by sex and absolute rate differences between men and women | 3 |
| **Appendix 6.** Incidence rates of probable or possible in-hospital delirium | 3 |
| **Appendix 7.** Incidence rates of in-hospital delirium among participants residing in nursing homes at baseline (n=631) | 4 |
| **Appendix 8.** Incidence rates of in-hospital delirium excluding participants with dementia in HUNT4 70+ | 4 |
| **Appendix 9.** Delirium ascertainment and subtype classification | 4 |
| **Appendix 10.** DSM-5 diagnostic criteria for delirium | 6 |
| **Appendix 11.** Inter-rater reliability assessment of delirium classifications | 6 |
| **Appendix 12.** Completed STROBE checklist for cohort studies | 7 |
| **References** | 10 |

**Appendix 1. Characteristics of hospital admissions after HUNT4 70+, overall and by delirium status.**

|  | **All admissions** | **Probable delirium** | **Possible delirium** | **F05 coded among probable delirium** |
| --- | --- | --- | --- | --- |
| **Total** | 19 272 | 2370 (12.3) | 1833 (9.5) | 283 (11.9) |
|  |  |  |  |  |
| **Admission type** |  |  |  |  |
| Acute | 14 770 (76.6) | 2176 (14.7) | 1629 (11.0) | 264 (12.1) |
| Elective | 4502 (23.4) | 194 (4.3) | 204 (4.5) | 19 (9.8) |
|  |  |  |  |  |
| **Department** |  |  |  |  |
| Medicine | 10 504 (54.5) | 1469 (14.0) | 1114 (10.6) | 216 (14.7) |
| Surgery | 7474 (38.9) | 820 (11.0) | 619 (8.3) | 62 (7.6) |
| Neurology | 456 (2.4) | 40 (8.8) | 53 (11.6) | 2 (5.0) |
| Other | 838 (4.4) | 41 (4.9) | 47 (5.6) | 3 (7.3) |
|  |  |  |  |  |
| **Illness severity (peak NEWS2 score)** |  |  |  |  |
| 0–2 | 8525 (44.2) | 445 (5.2) | 553 (6.5) | 48 (10.8) |
| 3–5 | 5095 (26.4) | 733 (14.4) | 583 (11.4) | 94 (12.8) |
| 6–8 | 2148 (11.2) | 563 (26.2) | 337 (15.7) | 64 (11.4) |
| ≥9 | 887 (4.6) | 391 (44.1) | 173 (19.5) | 45 (11.5) |
| Missing | 2617 (13.6) | 238 (9.1) | 187 (7.2) | 32 (13.5) |
|  |  |  |  |  |
| **Age at admission (years)** |  |  |  |  |
| 70–74 | 2897 (15.0) | 181 (6.3) | 159 (5.5) | 15 (8.3) |
| 75–79 | 6198 (32.2) | 499 (8.1) | 442 (7.1) | 42 (8.4) |
| 80–84 | 4742 (24.6) | 547 (11.5) | 498 (10.5) | 70 (12.8) |
| 85–89 | 3305 (17.1) | 629 (19.0) | 368 (11.1) | 85 (13.5) |
| ≥90 | 2130 (11.1) | 514 (24.1) | 366 (17.2) | 71 (13.8) |
|  |  |  |  |  |
| **Length of stay (days)** |  |  |  |  |
| 0–2 | 9054 (47.0) | 444 (4.9) | 570 (6.3) | 50 (11.3) |
| 3–5 | 6020 (31.2) | 776 (12.9) | 678 (11.3) | 93 (12.0) |
| 6–9 | 2463 (12.8) | 553 (22.5) | 312 (12.7) | 61 (11.0) |
| ≥10 | 1735 (9.0) | 597 (34.4) | 273 (15.7) | 79 (13.2) |

Values are n (%) unless otherwise specified. Percentages in the delirium columns represent the proportion with delirium within each category. Hospital transfers occurring on the same day were merged and treated as a single admission. Admission type and department were defined according to the first admission.

**Appendix 2. Hospital admission rates after HUNT4 70+, by baseline age group and sex.**

| **Age group** | **Men** | **Women** | **Total** |
| --- | --- | --- | --- |
| 70–74 | 328.6 | 259.3 | 292.8 |
| 75–79 | 422.3 | 356.2 | 386.8 |
| 80–84 | 534.6 | 433.3 | 477.9 |
| 85–89 | 669.6 | 574.5 | 607.9 |
| ≥90 | 682.1 | 536.0 | 583.2 |
| **70+ total** | **416.6** | **356.2** | **383.7** |

Values are incidence rates per 1000 person-years. Admissions were counted from the date of participation in HUNT4 70+ until 31 December 2023. Hospital transfers occurring on the same day were merged and treated as a single admission.

**Appendix 3. First events during follow-up (delirium, death before delirium, or censoring) by age group.**

| **Age group at baseline** | **Delirium** | **Death before delirium** | **Censored** |
| --- | --- | --- | --- |
| 70–74 | 299 (7.3) | 290 (7.1) | 3490 (85.6) |
| 75–79 | 327 (12.4) | 365 (13.9) | 1941 (73.7) |
| 80–84 | 352 (21.8) | 362 (22.4) | 902 (55.8) |
| 85–89 | 328 (34.1) | 373 (38.8) | 261 (27.1) |
| ≥90 | 201 (30.2) | 428 (64.3) | 37 (5.6) |
| **70+ total** | **1507 (15.1)** | **1818 (18.3)** | **6631 (66.6)** |

Values are n (%). Percentages are row percentages.

**Appendix 4. Distribution of delirium subtypes (by sex and overall), and F05-status for admissions classified as probable delirium.**

|  | **Male** | **Female** | **Total** | **F05 coded among probable delirium** |
| --- | --- | --- | --- | --- |
| **Delirium subtypes** |  |  |  |  |
| Hypoactive delirium | 433 (32.7) | 518 (42.8) | 951 (37.5) | 55 (5.8) |
| Hyperactive delirium | 478 (36.1) | 375 (31.0) | 853 (33.7) | 134 (15.7) |
| Mixed delirium | 327 (24.7) | 255 (21.1) | 582 (23.0) | 75 (12.9) |
| Not classified | 86 (6.5) | 62 (5.1) | 148 (5.8) | 23 (15.5) |

Values are n (%). Hospital transfers occurring the same day are treated as different admissions to preserve episode-level subtype classification. Totals therefore differ from tables where same-day transfers were merged.

**Appendix 5. Age-specific incidence rates of probable in-hospital delirium by sex and absolute rate differences (men minus women), per 1000 person-years.**

| **Age group** | **Men** | **Women** | **Rate difference** | **95% CI** |
| --- | --- | --- | --- | --- |
| 70–74 | 14.7 | 8.3 | 6.4 | 2.2–10.7 |
| 75–79 | 22.2 | 13.6 | 8.6 | 4.6–12.6 |
| 80–84 | 39.7 | 27.7 | 12.0 | 4.7–19.3 |
| 85–89 | 80.6 | 61.9 | 18.7 | 3.9–33.4 |
| ≥90 | 121.8 | 102.9 | 18.9 | –7.3–45.1 |

**Appendix 6. IPW-weighted incidence rates per 1000 person-years (95% CI) of in-hospital delirium (probable and probable + possible) by sex and age group.**

| **Age group** | **Probable** | **Probable + possible** |
| --- | --- | --- |
| **Men** |  |  |
| 70–74 | 14.7 (11.7–18.7) | 25.0 (20.9–30.0) |
| 75–79 | 22.2 (19.2–25.8) | 35.2 (31.3–39.7) |
| 80–84 | 39.7 (34.3–46.2) | 63.7 (56.7–71.9) |
| 85–89 | 80.6 (69.5–93.9) | 111.2 (97.6–127.0) |
| ≥90 | 121.8 (101.8–146.6) | 178.2 (152.9–208.1) |
| **70+ total** | **36.2 (33.6–39.0)** | **54.6 (51.4–58.0)** |
|  |  |  |
| **Women** |  |  |
| 70–74 | 8.3 (6.2–11.3) | 15.6 (12.7–19.6) |
| 75–79 | 13.6 (11.5–16.3) | 25.5 (22.4–29.1) |
| 80–84 | 27.7 (23.7–32.5) | 48.3 (42.8–54.6) |
| 85–89 | 61.9 (54.1–71.2) | 94.3 (84.1–106.0) |
| ≥90 | 102.9 (90.0–118.1) | 167.6 (149.9–187.6) |
| **70+ total** | **30.6 (28.5–32.9)** | **50.1 (47.3–53.1)** |
|  |  |  |
| **Total** |  |  |
| 70–74 | 11.4 (9.5–13.8) | 20.2 (17.6–23.3) |
| 75–79 | 17.7 (15.8–19.8) | 30.1 (27.6–32.8) |
| 80–84 | 33.0 (29.7–36.8) | 55.1 (50.7–60.1) |
| 85–89 | 69.2 (62.6–76.6) | 100.9 (92.5–110.1) |
| ≥90 | 109.0 (97.8–121.6) | 171.0 (156.3–187.4) |
| **70+ total** | 33.1 (31.5–34.9) | **52.1 (50.0–54.3)** |

**Appendix 7. Crude incidence rates of in-hospital delirium among participants residing in nursing homes at baseline (n=631), per 1000 person-years.**

| **Group** | **Person-years (events)** | **Incidence rate** | **95% CI** |
| --- | --- | --- | --- |
| **Sex** |  |  |  |
| Male | 354 (57) | 161.2 | 124.3–209.0 |
| Female | 915 (92) | 100.6 | 82.0–123.4 |
|  |  |  |  |
| **Age group** |  |  |  |
| 70–74 | 67 (10) | 149.5 | 80.4–277.9 |
| 75–79 | 147 (22) | 150.0 | 98.8–227.8 |
| 80–84 | 238 (26) | 109.4 | 74.5–160.6 |
| 85–89 | 294 (33) | 112.4 | 79.9–158.1 |
| ≥90 | 523 (58) | 110.8 | 85.7–143.4 |
|  |  |  |  |
| **All participants** | 1268 (149) | 117.5 | 100.0–138.0 |

**Appendix 8. IPW-weighted incidence rates of in-hospital delirium excluding participants with dementia in HUNT4 70+ (n=8394), per 1000 person-years.**

| **Age group** | **Incidence rate** | **95% CI** |
| --- | --- | --- |
| **Men** |  |  |
| 70–74 | 12.1 | 9.3–16.0 |
| 75–79 | 18.3 | 15.6–21.7 |
| 80–84 | 32.9 | 27.8–39.1 |
| 85–89 | 61.8 | 51.4–74.6 |
| ≥90 | 96.3 | 76.5–122.3 |
| 70+ total | 27.8 | 25.5–30.4 |
| **Women** |  |  |
| 70–74 | 6.9 | 4.9–9.8 |
| 75–79 | 12.4 | 10.3–15.1 |
| 80–84 | 22.2 | 18.5–26.9 |
| 85–89 | 49.4 | 41.8–58.8 |
| ≥90 | 99.7 | 84.5–118.2 |
| 70+ total | 24.1 | 22.1–26.3 |
| **Total** |  |  |
| 70–74 | 9.4 | 7.7–11.7 |
| 75–79 | 15.2 | 13.4–17.2 |
| 80–84 | 27.0 | 23.9–30.7 |
| 85–89 | 54.3 | 48.0–61.7 |
| ≥90 | 98.5 | 86.1–113.1 |
| **70+ total** | **25.8** | **24.3–27.4** |

**Appendix 9. Delirium ascertainment and motor subtype classification**

Delirium was identified by retrospective chart review using DSM-5 criteria, as described in the main text. The criteria were applied based on recorded symptoms and signs in the electronic health records (EHRs). Delirium status was classified as probable (all DSM-5 criteria fulfilled), possible (three or four criteria fulfilled with overall documentation supporting delirium), or no delirium (all remaining admissions). If three criteria were fulfilled, but the symptoms were considered more likely explained by another neurocognitive disorder alone, the admission was classified as no delirium. For example, patients with an epileptic seizure could fulfill criteria A, B, and C, but if the documented symptoms were considered fully explained by the seizure and there was no additional documentation supporting delirium, the admission was classified as no delirium despite fulfillment of three DSM-5 criteria. Full DSM-5 criteria are shown in Appendix 10.

We accessed the EHR system covering three hospitals in Central Norway (Levanger Hospital, Namsos Hospital, and St. Olav’s University Hospital). All available records were reviewed, including notes from physicians, nurses, and other health professionals, observation charts, and discharge summaries.

Given variability in the documentation of delirium-related symptoms, a keyword-based search strategy was not considered appropriate, and all records were therefore reviewed manually in full.

**Operationalisation of DSM-5 criteria**
To account for limitations in clinical documentation, two predefined operational clarifications were applied. First, to better capture cases of severe hypoactive delirium, criterion C (cognitive disturbance) was considered fulfilled when markedly reduced consciousness (excluding coma) was documented and the patient was explicitly described as too somnolent to assess cognitive function, provided that the remaining DSM-5 criteria were clearly documented. Second, altered awareness was accepted as the primary indicator of criterion A (disturbance in attention and awareness) when explicit documentation of impaired attention was lacking, reflecting the limited routine documentation of formal attention testing in medical records, consistent with previous chart-based delirium studies [1].

Criterion E was not recorded as a separate eCRF item but was operationally assumed to be fulfilled for all included somatic hospital admissions, given the clinical context of hospitalisation.

**Motor subtype classification**
After completion of data collection, delirium episodes were classified into motor subtypes based on responses to three items in the electronic case report form (eCRF):

1. **Agitated or restless? (yes / no / possible)**
2. **Somnolent or drowsy? (yes / no / possible)**
3. **Both hypo- and hyperactive symptoms? (yes / no / possible)**

Episodes were first classified as mixed delirium if question 3 was answered yes. Remaining episodes were classified as hyperactive or hypoactive delirium according to responses (yes) to questions 1 and 2, respectively. Responses coded as possible were not considered definitive. Motor subtype could not be determined in a minority of episodes, primarily reflecting inconsistent documentation of motor symptoms.

**Electronic case report form (eCRF)**
The eCRF used for data collection is provided in English translation below.

| **Item** | **Response Options** |
| --- | --- |
| Hospital | Levanger / Namsos / St. Olav |
| Admission date | dd MM yyyy |
| Discharge date | dd MM yyyy |
| Admission type | Acute / Elective |
| Department | Medicine / Surgery / Neurology / Other |
| Maximum NEWS2 score during admission | 0 – 20 (blank if NEWS2 not recorded) |
| Delirium ICD-10 coded (F05) | Yes / No |
| Main ICD-10 diagnosis for hospitalisation | Free text (ICD-10 code) |
| A – Awareness: Altered consciousness? | Yes / No / Possible |
| Agitated or restless? | Yes / No / Possible |
| Somnolent or drowsy? | Yes / No / Possible |
| Mixed hyper- and hypoactive symptoms? | Yes / No / Possible |
| A – Attention: Inattention? | Yes / No / Possible |
| B – Acute change in mental status? | Yes / No / Possible |
| B – Fluctuating course during admission? | Yes / No / Possible |
| C – Cognitive impairment? | Yes / No / Possible |
| Disorientation? | Yes / No / Possible |
| Memory problems? | Yes / No / Possible |
| Language difficulties? | Yes / No / Possible |
| Inappropriate behaviour? | Yes / No / Possible |
| Hallucinations? | Yes / No / Possible |
| Delusions? | Yes / No / Possible |
| D – Not better explained by known neurocognitive disorder | Yes / No / Possible |
| **Delirium status based on records** | **No delirium / Possible delirium / Probable delirium** |
| *Who described delirium?* | *Physician / Nurse / Physio or OT / Multiple sources* |
| *Time of day when delirium was described* | *Only daytime / Only evening / Only night / Multiple shifts* |
| *Is the word “delirium” used in records?* | *Yes / No* |
| *Or “acute confusion” or similar used?* | *Yes / No* |
| Delirium present preoperatively? | Yes / No |
| Delirium present postoperatively? | Yes / No |
| General comment | Free text |

**Appendix 10. DSM-5 diagnostic criteria for delirium** [2]

**Diagnostic criteria for delirium in DSM-5**

A. Disturbance in attention and awareness.

B. The disturbance develops over a short period of time (usually hours to a few days), represents an acute change from baseline attention and awareness, and tends to fluctuate in severity during the course of a day.

C. An additional disturbance in cognition (e.g. memory deficit, disorientation, language, visuospatial ability, or perception).

D. The disturbance in Criteria A and C are not better explained by a pre-existing, established or evolving neurocognitive disorder and do not occur in the context of a severely reduced level of arousal such as coma.

E. There is evidence from the history, physical examination or laboratory findings that the disturbance is a direct physiological consequence of another medical condition, substance intoxication or withdrawal, or exposure to a toxin, or is due to multiple etiologies.

**Appendix 11. Inter-rater reliability assessment of delirium classifications**

To evaluate the reliability of delirium classifications based on chart review, two rounds of inter-rater reliability (IRR) assessments were conducted.

In the first round (October–November 2024), 52 admissions were randomly selected from all eligible acute hospital admissions between 2006 and 2023 with a length of stay of at least 2 days. Sampling was stratified by age at admission, with one quarter of admissions drawn from each of the following age groups: 60–69, 70–79, 80–89, and ≥90 years. The raters were four physicians and four medical students.

In the second round (April 2025), 30 admissions were randomly selected from all eligible acute hospital admissions between 2006 and 2023 with a length of stay of at least 3 days (range 3–20). Sampling was stratified by age at admission, with one third of admissions drawn from each of the following age groups: 70–79, 80–89, and ≥90 years. The raters were two physicians (the same as in the first round), one nurse, and two medical students.

At the completion of the first IRR assessment on 22 November 2024, 7.4% of the planned reviews in the wider HUNT delirium project had been completed. At the completion of the second assessment on 14 April 2025, 37.3% had been completed. Thus, 62.7% of the reviews were completed after the second assessment.

In both assessments, all raters independently reviewed the same set of hospital admissions and classified delirium status into three categories: no delirium, possible delirium, or probable delirium.

Because the diagnostic categories represent an ordinal scale, agreement statistics were calculated using linear weights. Weighted Fleiss’ kappa was used as the primary measure of agreement and is the statistic reported in the main article and the table below. Additional agreement statistics, including the Brennan–Prediger coefficient, Gwet’s AC, and Krippendorff’s alpha were also calculated, but are not presented here.

Analyses were performed in Stata version 18 using the *kappaetc* command with the option *wgt(linear)* to apply linear weights.

**Inter-rater reliability of delirium classifications**

|  | **Admissions reviewed** | **Raters** | **Percent agreement** | **Weighted Fleiss’ kappa** | **95% CI** |
| --- | --- | --- | --- | --- | --- |
| **Round 1** | 52 | 8 | 89.7% | 0.65 | 0.52–0.78 |
| **Round 2** | 30 | 5 | 91.0% | 0.80 | 0.69–0.91 |

**Appendix 12. STROBE Statement—Checklist of items that should be included in reports of *cohort studies***

|  | **Item No** | **Recommendation** | **Location in manuscript** |
| --- | --- | --- | --- |
| **Title and abstract** | 1 | (*a*) Indicate the study’s design with a commonly used term in the title or the abstract | **Title:** population-based  **Abstract** (Methods): population-based cohort study |
|  |  | (*b*) Provide in the abstract an informative and balanced summary of what was done and what was found | **Abstract** (Background, Methods, Results, Conclusions) |
| **Introduction** | | |  |
| Background/rationale | 2 | Explain the scientific background and rationale for the investigation being reported | **Introduction** (paragraphs 1–4) |
| Objectives | 3 | State specific objectives, including any prespecified hypotheses | **Introduction** (final paragraph) |
| **Methods** | | |  |
| Study design | 4 | Present key elements of study design early in the paper | **Methods** – Study design and participants (first paragraph) |
| Setting | 5 | Describe the setting, locations, and relevant dates, including periods of recruitment, exposure, follow-up, and data collection | **Methods** – Study design and participants, delirium assessment, follow-up period, data sources |
| Participants | 6 | (*a*) Give the eligibility criteria, and the sources and methods of selection of participants. Describe methods of follow-up | **Methods** – Study design and participants Figure 1 (flow chart) |
|  |  | (*b*) For matched studies, give matching criteria and number of exposed and unexposed | Not applicable |
| Variables | 7 | Clearly define all outcomes, exposures, predictors, potential confounders, and effect modifiers. Give diagnostic criteria, if applicable | **Methods** – Delirium assessments (outcome definition, diagnostic criteria)  **Methods** – Study design and participants (covariates)  **Methods** – Statistical analysis (handling of variables) |
| Data sources/ measurement | 8* | For each variable of interest, give sources of data and details of methods of assessment (measurement). Describe comparability of assessment methods if there is more than one group | **Methods** – Delirium assessments  **Methods** – Study design and participants |
| Bias | 9 | Describe any efforts to address potential sources of bias | **Methods** – Delirium assessments (misclassification bias)  **Methods** – Statistical analysis (non-participation bias, inverse probability weighting) |
| Study size | 10 | Explain how the study size was arrived at | **Methods** – Study design and participants; Figure 1 (flow chart) |
| Quantitative variables | 11 | Explain how quantitative variables were handled in the analyses. If applicable, describe which groupings were chosen and why | **Methods** – Statistical analysis (age groups and handling of time variables) |
| Statistical methods | 12 | (*a*) Describe all statistical methods, including those used to control for confounding | **Methods** – Statistical analysis (incidence rate estimation, inverse probability weighting, and Poisson models for absolute rate differences) |
|  |  | (*b*) Describe any methods used to examine subgroups and interactions | **Methods** – Statistical analysis (analyses by age groups and sex) |
|  |  | (*c*) Explain how missing data were addressed | **Methods** – Statistical analysis (handling of missing education data)  **Results –** Table 1 |
|  |  | (*d*) If applicable, explain how loss to follow-up was addressed | **Methods** – Statistical analysis (censoring) |
|  |  | (*e*) Describe any sensitivity analyses | **Methods** – Statistical analysis (sensitivity analyses) |
| **Results** | | |  |
| Participants | 13* | (a) Report numbers of individuals at each stage of study—eg numbers potentially eligible, examined for eligibility, confirmed eligible, included in the study, completing follow-up, and analysed | **Methods** – Study design and participants (19 403 invited, 9956 participated)  **Results** (paragraphs 1–3)  Figure 1 (flow chart) |
|  |  | (b) Give reasons for non-participation at each stage | Reasons for non-participation in HUNT4 70+ were not available  **Methods** – Study design and participants |
|  |  | (c) Consider use of a flow diagram | Figure 1 (flow chart) |
| Descriptive data | 14* | (a) Give characteristics of study participants (eg demographic, clinical, social) and information on exposures and potential confounders | **Results** – Table 1 |
|  |  | (b) Indicate number of participants with missing data for each variable of interest | **Results** – Table 1 |
|  |  | (c) Summarise follow-up time (eg, average and total amount) | **Results** (first paragraph) |
| Outcome data | 15* | Report numbers of outcome events or summary measures over time | **Results** (paragraph 4)  **Results –** Table 2 |
| Main results | 16 | (*a*) Give unadjusted estimates and, if applicable, confounder-adjusted estimates and their precision (eg, 95% confidence interval). Make clear which confounders were adjusted for and why they were included | **Results** (paragraph 4–5)  **Results** – Table 2 (incidence rates) |
|  |  | (*b*) Report category boundaries when continuous variables were categorized | **Results –** Table 1 (frailty categories)  **Results –** Table 2 (age groups) |
|  |  | (*c*) If relevant, consider translating estimates of relative risk into absolute risk for a meaningful time period | Not applicable (absolute incidence rates reported) |
| Other analyses | 17 | Report other analyses done—eg analyses of subgroups and interactions, and sensitivity analyses | **Results** (sensitivity analyses)  **Supplementary data** (Appendices 5–8) |
| **Discussion** | | |  |
| Key results | 18 | Summarise key results with reference to study objectives | **Discussion** (first paragraph) |
| Limitations | 19 | Discuss limitations of the study, taking into account sources of potential bias or imprecision. Discuss both direction and magnitude of any potential bias | **Discussion** (limitations section) |
| Interpretation | 20 | Give a cautious overall interpretation of results considering objectives, limitations, multiplicity of analyses, results from similar studies, and other relevant evidence | **Discussion** |
| Generalisability | 21 | Discuss the generalisability (external validity) of the study results | **Discussion** (strengths and generalisability paragraphs) |
| **Other information** | | |  |
| Funding | 22 | Give the source of funding and the role of the funders for the present study and, if applicable, for the original study on which the present article is based | **Declaration of Sources of Funding** |

*Give information separately for exposed and unexposed groups.

**Note:** The STROBE checklist for cohort studies was downloaded from the STROBE Statement website and is based on the STROBE Statement [3].

**References**

1.	Geriatric Medicine Research Collaborative. Retrospective delirium ascertainment from case notes: a retrospective cohort study. *BMJ Open* 2021;**11**(5):e042440. https://doi.org/10.1136/bmjopen-2020-042440.

2.	American Psychiatric Association. *Diagnostic and Statistical Manual of Mental Disorders*. Fifth Edition, Arlington, VA: American Psychiatric Publishing, 2013.

3.	von Elm E, Altman DG, Egger M *et al.* The Strengthening the Reporting of Observational Studies in Epidemiology (STROBE) statement: guidelines for reporting observational studies. *J Clin Epidemiol* 2008;**61**(4):344–9. https://doi.org/10.1016/j.jclinepi.2007.11.008.
